# Review of *Mycobacterium marinum* Infection Reported From Iran and Report of Three New Cases With Sporotrichoid Presentation

**DOI:** 10.5812/ircmj.10120

**Published:** 2014-02-05

**Authors:** Farhang Babamahmoodi, Abdolreza Babamahmoodi, Babak Nikkhahan

**Affiliations:** 1 Infectious Diseases and Tropical Medicine Department, Antimicrobial Resistance Research Center, Mazandaran University of Medical Sciences, Sari, IR Iran; 2 Health Management Research Center, Baqiyatallah University of Medical Sciences, Tehran, IR Iran

**Keywords:** *Mycobacterium marinum*, Granuloma, Iran

## Abstract

**Introduction:**

Mycobacterium marinum infection is the most common nontuberculous mycobacterial skin lesions. It results from skin injury and contact with contaminated water, fish, or shellfish; its infections have low frequency, nonspecific symptoms and lack of specific identification methods that can alter correct diagnosis.This study designed about cases that reported from Iran and comparing their presentation and clinical sign and symptom and outcome.

**Case Presentation:**

We find and evaluate three cases that have been reported in indexing sites (PubMed, Google scholar and Iranian indexing databases) since 1980 till end of 2012. Using combinations of the following keywords: “*Mycobacterium marinum*,” “Iran”, “atypical mycobacterium”, “Sporotrichoid presentation” and “fish tank granuloma”. Three new cases also described that infected with this organism and had Sporotrichoid presentation in 2012 in a referral hospital in north of Iran.

**Conclusions:**

Totally we evaluate six patients. Source of infection in all cases were aquarium and four of six cases were male (66.6%). Occurrence to treatment interval were between one month to one year (mean 3.07 months). Infection site in all of them were hands and dominantly in right hand (66.6 % of cases ) and 83.3 % of them had Sporotrichoid presentation and all of the patients finally cured. The only cause of infection with Mycobacterium marinum in Iran is aquarium and its presence in homes and offices increased during these years. Health workers and people should be informed and warned about this disease.

## 1. Introduction

*Mycobacterium marinum* formerly called *M. balnei* is a free-living bacterium which causes opportunistic infections in humans; causing chronic cutaneous lesions and in some cases deeper infections such as Tenosynovitis, septic arthritis and rarely osteomyelitis (1, 2). Aronson isolated this organism in 1926 from a fish and the first case of *M. marinum* skin infection was reported in 1951 (3). It is atypical mycobacteria which are acid-fast, facultative pathogens or saprophytes. Most infections are acquired in swimming pools, beaches, rivers, and lakes, or by cleaning aquariums (4). Vectors of infection include fresh- or saltwater fish, snails, shellfish, dolphins, and water fleas (5). Infection may be acquired by direct inoculation through injured skin in an aquatic environment. Risk factors include history of skin injuries and water/fish related hobbies or occupations (1). The disease begins as a violaceous papule or nodule. It can also present as a psoriasiform or verrucous plaque, usually on the hands, feet, elbows or knees, at the site of trauma, about 2 to 3 weeks after inoculation. These may be solitary but are often multiple and occasionally sporotrichoid spread occurs. Three types of lesions are recognized: a solitary granulomatous verrucous papule that may occasionally ulcerate and show purulent discharge; ascending lymphatic sporotrichoid lesions; and rare cutaneous disseminated lesions, which occur frequently in immunosuppressed patients. Lesions are painful in less than one half of cases (4).

The lesions may ulcerate or frequently heal spontaneously within 1 to 2 years, with residual scarring. Sometimes, penetration to underlying structures (bursae, joints) may occur (2). Regional lymph nodes are, as a rule, not involved and lymphadenopathy is rare and typically mild, and systemic symptoms are unusual .Occasionally, the lesions are suppurative rather than granulomatous which may be multiple in immune suppressed hosts (1) in the United States annual prevalence of *M. marinum* infection is 0.27 in each 100000 person (6) and delay in diagnosis is considered more a rule than an exception in *M. marinum* infection (7). The clinical presentation is often insidious and nonspecific and key data may be missed; furthermore rarity of the infection, the lack of clinical suspicion, diverse manifestations and a failure to elicit the history of aquatic exposure will help to this process and the diagnosis is often delayed (7, 8). *M. marinum* infections can cause significant morbidity, including loss of joint mobility due to osteomyelitis and even amputation of the affected extremities. A proactive approach to obtain a biopsy for histopathological and microbiological diagnosis is advised. Sometimes, culture of *M. marinum* is negative but the diagnosis is still made on physical signs supported by typical histological findings (8). Various DNA-based techniques have been used to classify mycobacteria. A prolonged course of antibiotic therapy is curative in most superficial cases. Anti-mycobacterium treatment should be started promptly. The combined use of Rifampicin, Ethambutol, and Clarithromycin appears to be effective. Adjunctive surgical debridement is sometimes indicated in extensive and deep infections (1-8).

The main differential diagnosis of *M. marinum* cutaneous granuloma is sporotrichosis. A foreign body granuloma should be considered. Some other clinical entities possibly mimicking *M. marinum* infection include cutaneous leishmaniasis, psoriasis, verrucous lichen planus, verruca vulgaris, iodine and bromine granulomas, sarcoidosis, syphilis, gout, and chronic pyogenic infections. In addition, *Pasteurella tularensis*, *Scopulariopsis blochi*, and *Nocardia brasiliensis* infections must be considered in the differential diagnosis (9). We report three new cases of this infection with *Sporotrichoid* presentation and along with three cases that we found in literature review about the cases reported from Iran. We conducted a systematic search of the on-line databases Web of Knowledge, PubMed, Google Scholar and Iranian indexing database (magiran, ISC and Iranmedex) using combinations of the following keywords: “*Mycobacterium marinum*”, “Iran”, “atypical mycobacterium”, “Sporotrichoid presentation” and “fish tank granuloma,”. Reference lists of relevant articles were also searched. The results were assessed for inclusion using the publication title and abstract. Restrictions regarding publication dates were applied (between 1980 till 2012). This study was done in Iran, Qaemshahr city in north of Mazandaran province. All new cases that reported in this paper have been referred to Razi's hospital of mentioned city.

## 2. Case Presentation

### 2.1. Case One

**Figure 1.**
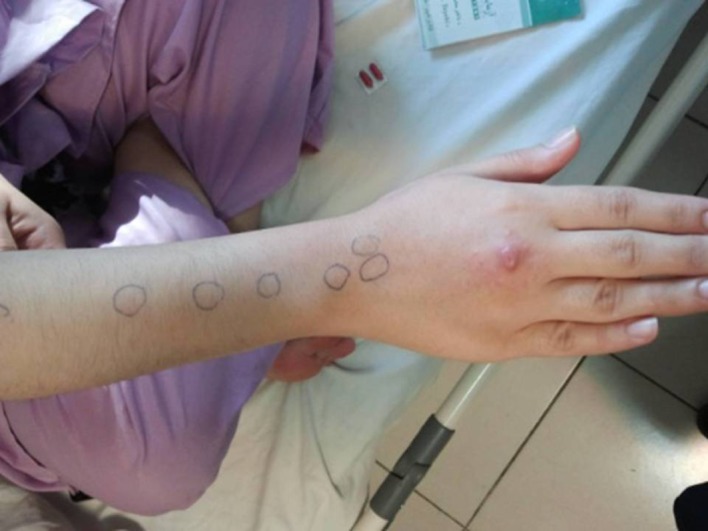
Patients Right Hand

The patient is a 29 year old lady, married and house keeper .she came to our office with an erythematous nodule on back of her right hand and multiple painless subcutaneous nodules that are in linear pattern spread toward the elbow. The patient had a precedent history of a penetrating injury When she was cleaning the fish tank in her house (one centimeter length) just at the site that first nodule appeared. One week later she developed local redness with linear spread toward the elbow at the same hand. She received 200 mg Doxicycllin per day for 14 days. Although redness decreased but nodules size did not have any changes and after that multiple subcutaneous nodules appeared (Figure 1). In physical examination her general condition was good, vital signs were stable and had not any significant problem but in her right hand in 3rd metacarpofalingial region had a 1 cm superficial wound with an erythematous nodule and multiple satellite papules around it. There were 7 non tender approximately 1.5 cm nodules between wrist and elbow. There were no epitrochlear or axillary lymphadenopathy. She had complaint about itching at the wound site.

In past medical history she had cesarean section 7 month ago and hypothyroidism since one year ago that she was on levothyroxin. She had not significant diseases in her family history. On laboratory investigations, the blood, urine and stool were all within normal limits. Gram staining and smear for Leishman-Donovan bodies was negative. Hepatitis B surface antigen and HIV screening were negative and PPD test was positive (20 mm). Chest X-ray was normal. In Gram Stain Gram positive bacilli was seen with many PMN and in Ziehl Nellson Stain Few Acid fast Elements were seen. Microscopy and direct KOH test for fungal spores were also negative. Bacterial culture of blood and wound secretion also had not any significant data. Histopathology of skin revealed acanthosis of the epidermis with dermal lymphocytic infiltrate and few Langhan’s giant cells while fine needle aspiration cytology showed a tuberculous picture. Diagnosis was *M. marinum* lymphangitis and the patient were treated with the following medications; Ethambotul 800 mg/daily, Rifampicin 600 mg/daily and Doxicycllin 100 mg/bid. After four month patent had not any sign and symptom and treatment were discontinued after 6 months.

### 2.2. Case Two

A 32-year-old man from north of Iran who worked at a supermarket was presented with a history of painless, livid, verrucous nodular swelling and exudative erythematous lesions and pustules of the 5th finger of right hand. The first skin lesion had appeared on the extensor side of the finger 1.5 months ago. This case was treated with Cephalexin 500 mg/Q6H without any improvement. There were no systemic complaints and vital signs were stable. The patient mentioned an injury at the site of the first lesion. He did not have contact with domestic animals and never been in the tropics, but his family had owned an aquarium with tropical fish for the past 2 years and he cleaned it regularly. There were no risk factors for HIV infection. Family history of skin diseases was negative. Clinically, a long, painless, solid, livid, verrucous infiltrate 15 × 10 mm in diameter was localized on the extensor side of the right hand and Sporotrichoid pattern of distribution was obviously present. Histopathology examination of the lesions showed nonspecific inflammation to granuloma formation. Results of gram stain and routine culture from biopsy of skin lesions in both blood agar and McConckey agar were negative. In laboratory tests complete blood count and ESR were in normal range but PPD was 21 mm. A smear from the walls of the necrotic center when stained for acid-fast bacilli revealed organisms consistent with mycobacteria. The skin lesion was cultured for mycobacteria on Lowenstein-Jensen at 30 and 37˚C. Growth occurred after 12 days at 30˚C and did not grow at the incubation temperature of 37˚C. The results of the biochemical tests were indicative of *M. marinum*. On the basis of antibiogram, Treatment started with Ethambotul 800 mg/daily, Rifampicin 600 mg/daily and clarerithromycin. After 2.5 months all signs and symptoms disappeared and treatment were discontinued after 4 months.

### 2.3. Case Three

The patient was a single 28 year old man from Amol city in Iran .he was shopkeeper and since four weeks ago developed a single purple papule on bake side of his left hand. Lesion was painful but he did not have any complaint about itching or burning after some days the lesion changed to erythematous nodule and crusted ulcer. He did not have contact with domestic animals and never been in the tropical area and in his familial and medical history there were no significant problem. He reported irregular but multiple cleaning of aquarium during last year. Due to resistance to medication biopsy was done and acid fast bacilli was seen in culture and smear. The skin lesion was cultured for mycobacteria on Lowenstein-Jensen at 30˚C and 37˚C. Growth occurred after 12 days at 30˚C and did not grow at the incubation temperature of 37˚C. The results of the biochemical tests were indicative of *M. marinum*.

Histopathology examination of the lesions showed nonspecific inflammation to granuloma formation. Results of gram stain and routine culture from biopsy of skin lesions in both blood agar and McConckey agar were negative. In laboratory tests complete blood count and ESR were in normal range but PPD was 25 mm. On the basis of antibiogram, treatment started with clarerithromycin 500 mg/bid and after 3 months patient cured completely and treatment discontinued after 4 months (Table 1). 

**Table 1.** Summery of the New Patients Reported in This Paper

	Case 1	Case 2	Case 3
**Age, y**	29	32	28
**Sex**	female	male	Male
**Job **	House keeper	Supermarket worker	shopkeeper
**Presenting sign**	erythematous nodule on back of her right hand and multiple painless subcutaneous nodules that are in linear pattern spread toward the elbow Sporotrichoid pattern of distribution was obviously present	painless, livid, verrucous nodular swelling and exudative erythematous lesions and pustules a long, painless, solid, livid, verrucous infiltrate 15 × 10 mm in diameter was localized on the extensor side of the right hand and Sporotrichoid pattern of distribution was obviously present	Painful purple papule on bake side of his left hand erythematous nodule and crusted ulcer Sporotrichoid pattern of distribution was obviously present
**History**	penetrating injury when she was cleaning the fish tank in her house	Injury at the site of the first lesion. his family had owned an aquarium with tropical fish for the past 2 years and cleaned it regularly himself	irregular but multiple cleaning of aquarium during last year
**Location **	right hand in 3^rd^ metacarpofalingial region	5th finger of right hand	on bake side of his left hand
**Source of contact **	Aquarium	Aquarium	Aquarium
**Histopathology **	acanthosis of the epidermis with dermal lymphocytic infiltrate and few Langhan’s giant cells	nonspecific inflammation to granuloma formation	nonspecific inflammation to granuloma formation
**Libratory findings **	positive PPD test (20 mm); in Ziehl Nellson Stain Few Acid fast Elements were seen; KOH test for fungal spores were also negative	PPD was 21 mm; A smear from the walls of the necrotic center when stained for acid-fast bacilli revealed organisms consistent with mycobacteria; Results of the biochemical tests were indicative of *M. Marinum*	PPD was 25 mm; acid fast bacilli was seen in culture and smear; The biochemical tests were indicative of *M. Marinum*
**Medical history**	Levothyroxin since one year ago	No	No
**Treatment**	Ethambotul 800 mg/daily; Rifampicin 600 mg/daily and Doxicycllin 100 mg/bid	Ethambotul 800 mg/daily; Rifampicin 600 mg/daily and clarerithromycin	clarerithromycin 500 mg/bid
**Result**	Complete cure	Complete cure	Complete cure

### 2.4. M. Marinum in Iran

In the published data we searched and found only three reported cases of *M. marinum* infection from Iran till end of 2012. Golpour M et al. reported first case at 2007 and after that Hosseini Fard SM et al. and Alaeen AM et al. published two other cases (10-12) (Table 2). 

**Table 2.** Data About Cases Reported From Iran in Journals and Data Bases and Tree New Cases That We Report in This Paper

Author	Date of Report	age	Sex	Involved Part of Body	Source of Infection	Occurrence to Treatment Interval	Treatment	Duration of Treatment	Outcome	Clinical Presentation
**Golpour M**	2007	16	male	right hand	aquarium	1 year	Rifampin; Ethambutol	6 months	cure	sprotichoid bula
**Hosseini Fard SM**	2009	27	male	right hand	aquarium	2 months	Rifampin; ethambutol	6 months	cure	swelling and exudative erythematous lesions and pustules
**Alaeen AM**	2010	25	female	left hand -4th finger	aquarium	4 months	Doxycycline	2 months	cure	non tender nodules
**Babamahmoodi F **	2011	29	Female	right hand-3rd finger	aquarium	2 months	Rifampicin; Ethambutol Clarerithromycin	6 months	cure	Lesion at injury site and sprothrichoid pattern
**Babamahmoodi F**	2011	32	male	right hand-5th finger	aquarium	1.5 months	Rifampicin; Ethambutol Clarerithromycin	6 months	cure	sprothrichoid pattern
**Babamahmoodi F**	2011	28	male	Back of left hand	aquarium	1 month	Clarerithromycin	4 months	cure	sprothrichoid pattern

## 3. Discussion

Our report is 4th, 5th and 6th reports of *M. marinum* infection from Iran. Totally with these cases six cases of *M. marinum* reported from Iran. Patient's age range was between 16 to 32 year and 66.6 % of them were male and 100 % of lesions were on upper extremities that equally distributed on right and left side. Origin of infection in all of the cases was aquarium. From occurrence of diseases to beginning of the treatment minimum one month and maximum one year delay were present. In 66.6 % Sporotrichoid presentation reported and Rifampin and Ethambutol were the most frequent prescribed treatment after them Clarerythromycine and Doxycycline. Outcome was remarkable and 100 % of cases cured without any complication. Ghaemi EO et al. have done study for determination of the fish tank granuloma in Fishermen and *M. marinum* infection in south east Caspian Sea, north of Iran (13). Any suspected lesions in 387 subjects of Fishermen and 113 autopsy samples from gills of fish of Caviar were obtained and culture in Lowenstein Jensen medium. The mycobacterial species were determined by conventional biochemical tests. No fish tank granuloma was proved in human, but 11 (9.73 %) strains of Mycobacteria were isolated from Caviar fishes, that only two strains (1.76 %) belong with *M. marinum* (13).

Behrouznasab et al. in a study in Iran showed that *M. marinum* is major agent of swimming pool granuloma in their study a total of 58 paraffin tissue blocks were obtained and deoxyribonucleic acid (DNA) isolated the polymerase chain reaction (PCR) that was used to amplify the 16S rRNA gene (14). PCR amplification demonstrated the presence of *Mycobacterium* spp. in 18 blocks (31 %). Among these 18 blocks, 8 (44 %) positive for *M. marinum*, 33 (17 %) for M. ulcerans, 5 isolates (27 %) *M. fortuitum* and *M. chelonae*, 2 (12 %) *M. avium* (14). The average time from clinical presentation to correct diagnosis vary from 1 to 27 months with a mean interval of 7 months (15). In our study this time is less than 4 months. An extremely prolonged course of disseminated *M. marinum* infection, lasting 45 years, has also been reported (16). The diagnosis of *M. marinum* infection should be confirmed by histology and bacteriology (15), but these goals are sometimes difficult to achieve. Therefore, the diagnosis in practice is mostly based on memorized data, clinical and histological features, and response to therapy (17). *M. marinum* infections arise after skin trauma and after contact with contaminated water. Today, the infection is often aquarium-related; in 50 to 84 % of cases the affected are aquarium owners (3). In our study in 100 % of cases origin of infection were aquarium.

In most of the reported cases in the past decades, the upper limbs were affected, especially the fingers (7). Among aquarium owners, the hands are most commonly affected (17). In our study all of the cases involvement was on upper extremity. Sporotrichoid dissemination is possible in 20 to 40 % of infections (3). In our study 66.6 % of cases had Sporotrichoid presentation several cases have been reported in recent years (7). Infections related to swimming pools were very common before 1962. Due to improvement in disinfection and chlorination of swimming pool water, this source of infection is relatively uncommon nowadays and is believed to contribute to only 2.6 – 4.4 % of all infections (3, 4, 17). In one case series report by Aubry A. et al. in France from January 1, 1996, to December 31, 1998 they reported Sixty-three cases of *M. marinum* infection among them In 53 (84 %) of the patients, was related to fish tank exposure. The site of infection was mainly the upper limb (in 60 [95%] of the 63 patients), and infection was spread to deeper structures in 18 (29 %) of the patients. All patients were treated with antibiotics (median time, 3 (1/2) months), and 30 (48 %) underwent surgery. Various antibiotic regimens were prescribed, and the initial regimen was modified in 22 (35 %) of the patients. Clarithromycin, cyclines, and rifampin were the most commonly prescribed antibiotics. Cure was observed for 55 (87 %) of the patients. Failure was related to deep structure involvement (3 of 45 vs. 5 of 18 patients; P = 0.04) but not to any antibiotic regimen (18). Tsai HC et al. in a review article showed that in 14 studies, totally 166 cases were reported that 28 % of them were female and age range was between 4 years to 85 years and treatment duration had variation between one to 14 months. Incubation period ranged from a few hours to 8 years (mean 6.8 months) and presumed source of infection in 70 % of cases were aquarium or see products manipulation. All of them received antibiotics and 85 % of them cured and 10 % of them had treatment failure (19). Although this is the first study about this diseases in Iran but in this study we just used papers that indexed in web based data banks and there was not accessibility to non web based data banks and due to that may be missed some cases from Iran. According to our searches there is not any national data center that record *M. marinum* cases and these may have negative effects on this study. In conclusion, *M. marinum* infections are emerging infections related to fish tank amusement. Approximately all studies show the same epidemiological results. Due to severity of the cases with spread of infection, Knowledge about this rare condition is important and clinical attentiveness of *M. marinum* infection and its associated risk factors is important. So that physicians can avoid unnecessary diagnostic procedures and therapy can be initiated promptly. Funding a data bank for recording atypical tuberculosis cases in national and international level may be informative and useful and can help better planning for control of this rare condition.
